# Aortic Dilatation in Patients With Bicuspid Aortic Valve

**DOI:** 10.3389/fphys.2021.615175

**Published:** 2021-07-06

**Authors:** Jing Wang, Wenhui Deng, Qing Lv, Yuman Li, Tianshu Liu, Mingxing Xie

**Affiliations:** ^1^Department of Ultrasound Medicine, Union Hospital, Tongji Medical College, Huazhong University of Science and Technology, Wuhan, China; ^2^Clinical Research Center for Medical Imaging in Hubei Province, Wuhan, China; ^3^Hubei Province Key Laboratory of Molecular Imaging, Wuhan, China

**Keywords:** bicuspid aortic valve, aortic dilation, aneurysm, aortopathy, nature history

## Abstract

Bicuspid aortic valve (BAV) is the most common congenital cardiac abnormality. BAV aortic dilatation is associated with an increased risk of adverse aortic events and represents a potentially lethal disease and hence a considerable medical burden. BAV with aortic dilatation warrants frequent monitoring, and elective surgical intervention is the only effective method to prevent dissection or rupture. The predictive value of the aortic diameter is known to be limited. The aortic diameter is presently still the main reference standard for surgical intervention owing to the lack of a comprehensive understanding of BAV aortopathy progression. This article provides a brief comprehensive review of the current knowledge on BAV aortopathy regarding clinical definitions, epidemiology, natural course, and pathophysiology, as well as hemodynamic and clinically significant aspects on the basis of the limited data available.

## Introduction

Bicuspid aortic valve (BAV) disease is one of the most common congenital heart diseases, with a population incidence of about 0.5–2.0%, with male subjects the most commonly affected (Kong et al., 2017; Liu et al., 2018). The onset of BAV is insidious in the initial stage of life, but symptoms often manifest in adulthood and show considerable heterogeneity (Siu and Silversides, 2010). While the majority of patients develop complications such as aortic valve dysfunction and aortic dilatation (Siu and Silversides, 2010; Michelena et al., 2011), a few patients may remain asymptomatic for their whole life. Aortic dilatation can progress to aortic aneurysm or aortic dissection/rupture, threatening the life of BAV patients. Timely surgical intervention is the basic treatment strategy for aortic dilatation with BAV (Hiratzka et al., 2016). As the natural history of BAV disease is not completely understood, current surgical strategies remain controversial and come with high patient burden. The purpose of this review is to evaluate recent advances regarding management of BAV to provide better guidance on the basis of the limited data available.

## Definitions

A normal aortic valve consists of three nearly identical aortic valve cusps, each with a half-moon appearance. A BAV typically consists of the two leaflets of unequal size, in which two of the three leaflets fuse are the most common pattern. “Pure” BAV comprises two cusps of unequal size with no raphe, however, this is rarely seen. The heterogeneity of the spatial location of cusps or commissure(s) challenges the diagnosis of valve morphology.

The Sievers and Schmidtke’s classification based on surgical specimen is the most widely used classification for BAV. The classification system is composed of three categories and two supplementary categories. The three categories are: type 0 for valve with no raphe; type 1 for valve with one raphe; and type 2 for valve with two raphes. Within each category, the BAV phenotype is classified further based on two supplementary characteristics: spatial position of cusps (ap, anterior-posterior; lat, lateral) and functional status of the valve (I, insufficiency; S, stenosis; B, balanced valvular lesion; No, normal function). The Sievers and Schmidtke classification is based on the existence of raphe and the orientation of leaflet fusion, but does not include tricommissural and BAV consortium. Considering that the raphe(s) or the presence or absence of a third commissure could have an impact on the expansion and apposition of transcatheter aortic valve replacement (TAVR) prosthesis, Jilaihawi et al. (2016) proposed a novel morphological classification of TAVR-directed imaging of BAV aortic stenosis (AS), in combination with a rigorous computed tomography (CT) core-lab assessment. This typing system encourages a new valve typing method for BAV patients considering TAVR. Although anatomies differences in bicuspid and tricuspid aortic valve, TAVR for BAV stenosis indicates similar outcomes among 30-day and 1-year all-cause mortality, valve hemodynamics, and quality-of-life improvement to patient undergoing TAVR in tricuspid aortic stenosis (Forrest et al., 2020). It is worthy of further and extensive exploration in the future to establish a morphologic classification system that plays a major role both in preoperative imaging identification of morphological subtypes and risk stratification of postoperative complications.

**FIGURE 1:**
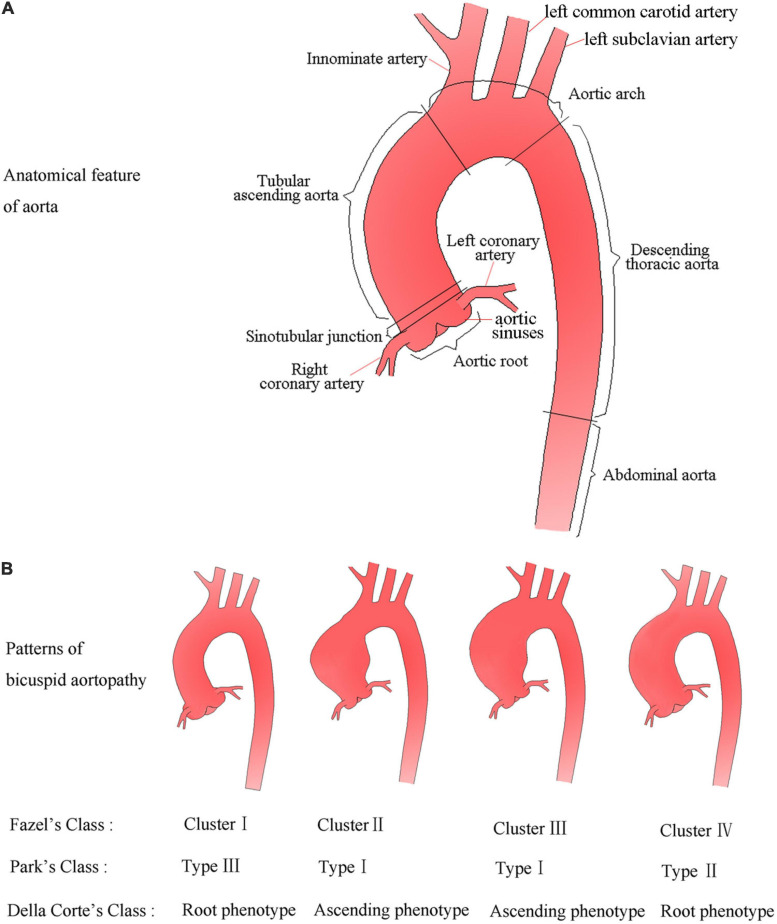
Anatomical feature of aorta and schematic diagram **(A)**, and possible patterns of aortic dilatation associated with BAV according to the three classifications **(B)**.

The aorta is a geometrically complex organ and is anatomically divided into five sections (Figure 1A). The Society for Vascular Surgery stratified the aortic diameter by sex and location of aorta based on CT or chest radiography, and proposed the normal thoracic aortic diameter range for adults (Johnston et al., 1991; Hiratzka et al., 2010a). In general, normal aortic diameters in healthy adults should not exceed 40 mm (Erbel et al., 2014). If the internal diameter of the local dilated aorta exceeds at least 50% of the expected normal diameter, it is defined as an aneurysm (or true aneurysm) (Hiratzka et al., 2010a). The aortic dimension is influenced by many factors such as age, sex, and body size; location of aortic segmental; imaging methods; and the specifications. Two-dimensional transthoracic echocardiography (2D-TTE) is a conventional method for measuring aortic diameter, and consensus is reached on the normal range of aortic diameter based on the age, sex, and body surface area (BSA) levels (Goldstein et al., 2015; Lang et al., 2015). Magnetic resonance imaging (MRI) as a method to measure aortic diameter will be discussed later in the manuscript. The upper limit of normal aortic diameter was defined as 2 SDs greater than the mean predicted diameter (Goldstein et al., 2015). The *Z* score means the number of SDs above or below the predicted mean normal diameter, is particularly useful for evaluating aortic dilatation in growing children. Taking into consideration the effects of increasing age and body size of developing children, an aortic diameter can be defined as aortic dilatation when the *z*-score is ≥2 (Goldstein et al., 2015).

The aortic dilatation classification has not been unified, and various classification models with descriptive value or prognostic potential have been proposed. Fazel et al. (2008) proposed four distinct patterns of aortic dilatation in BAV patients: cluster I, only aortic root involved; cluster II, only tubular ascending aorta involved; cluster III, tubular ascending aorta and transverse aortic arch involved; and cluster IV, diffuse aortic dilatation involving the aortic root, ascending aorta, and the proximal aortic arch. Park et al. (2011) from the Mayo Clinic proposed a classification scheme describing dilatation of the ascending aorta based on the potential effect of the general root phenotype on prognosis. The classification model includes three types except for the normal aorta, namely, type I: tubular ascending aorta enlargement with preservation of the STJ; type II: ectasia of the ascending aorta and root with effacement of the STJ; type III: enlargement confined to the root (Park et al., 2011). Della Corte et al. (2014) proposed the simplest classification for dividing the ascending aorta dilatation into two main phenotypes: aortic root phenotype (dilated dimensions, aortic sinuses > ascending aorta) and ascending phenotype (dilated dimensions, ascending aorta > aortic sinuses). Although simplistic, this classification system showed a potential prognostic value for aortic dilatation (Della Corte et al., 2014). Later studies have indicated non-negligible distinctions in embryonic origin of the tissue, circulating biomarkers, and functional imaging parameters between aortic sinuses and the ascending aorta (Cheung et al., 2012; Harmon and Nakano, 2013; Goudot et al., 2019b), which seemed to justify the rationality of Della Corte et al.’s classification. This is probably by far the simplest and most understandable classification model. For a better overview, patterns of bicuspid aortopathy are summarized in Figure 1B.

## Epidemiology

The reported prevalence of aortic dilatation in individuals with BAV is approximately 50% (Verma and Siu, 2014). The prevalence of tubular ascending aorta dilatation in BAV patients at ages < 30, 30–40, 40–50, 50–60, and >60 years has been reported to be 56, 74, 85, 91, and 88%, respectively, and the prevalence of aortic root dilatation in the same age groups were 48, 69, 52, 57, and 67%, respectively (Della Corte et al., 2007).

Men appear to have more frequent larger aortic dimensions and aortic dilatation than women (Andrei et al., 2015). The percentage of aortic dilatation was significantly higher in men with aortic dimensions between 40 and 44 mm (24% vs. 17%), and 45 and 49 mm (14% vs. 9%) than women (Kong et al., 2017). Men showed a higher frequency of aortic root dilatation (14.2% vs. 6.7%) and diffuse dilatation of the aorta (16.2% vs. 7.3%) than women (Kong et al., 2017).

Bicuspid aortic valve patients with AS showed an increase in the prevalence of dilatation in the tubular ascending aorta (Rossi et al., 2013; Roman et al., 2017), whereas the prevalence of the diffuse dilatation of the aorta (annulus, aortic root, or tubular ascending aorta) was increased in BAV patients with aortic regurgitation (AR; Roman et al., 2017). The prevalence of aortic dilatation also varies widely according to the valve phenotype. The prevalence of proximal aortic dilatation (aortic root and tubular ascending aorta, 79.0 and 79.6% respectively) was higher in type1 (Khoo et al., 2013; Li et al., 2019). The prevalence of dilatation of ascending aorta and proximal aortic arch was frequent in BAV patients with type-0 and AS (60.0%; Li et al., 2019). Aortic dilatation was more common in BAV patients with left-right coronary cusp fusion (L-R) than in those with right-non-coronary cusp fusion (R-N; Khoo et al., 2013).

## Natural Course

In healthy individuals, the growth rate of the aorta is about 0.9 mm in men and 0.7 mm in women for each decade of life. In BAV patients, the reported growth rate of the aorta varies from 0.2 to 1.9 mm per year (Davies et al., 2007; Detaint et al., 2014), with an annual growth rate of 0.76 mm (Etz et al., 2010; Guo et al., 2018). Variations in growth rates might be related to population-based data, statistical methods, assessment techniques, and timeliness, as well as heterogeneity of the disease. The initial diameter of the ascending aorta may result in different rates of dilatation (Park K. H. et al., 2017). The mean growth rate of an adult ascending aorta with a diameter of 40–44 mm, 45–49 mm, and at least 50 mm has been reported to be 0.3 ± 0.5 mm/year, 0.3 ± 0.5 mm/year, and 0.7 ± 0.9 mm/year, respectively (Park K. H. et al., 2017). All segments of the ascending aorta in adults with BAV were larger than in adults with tricuspid aortic valve (TAV).

The progression of aortic dilatation in BAV patients shows variation in many aspects. Della Corte et al. retrospectively analyzed a series of data from 133 adult outpatient BAV patients who were followed up by echocardiography to explore risk factors for rapid growth of the ascending aorta over time (Della Corte et al., 2013). They found a mean growth rate of 0.3 mm/year at the aortic root level (sinuses of Valsalva) and 0.6 mm/year at the ascending level; yet, growth was somewhat insignificant (mean, 0.09 mm/year) to negligible at the STJ (Della Corte et al., 2013). The aortic root phenotype often predicts more severe aortopathy and aortic dilatation progresses faster (>0.9 mm/year) at the level of the ascending aorta. However, patients with BAV with R-L and an ascending dilatation phenotype proved to be more stable and rarely showed fast growth (Della Corte et al., 2013). Though, another retrospective study found that R-L was a risk factor for faster aortic dilatation, with an annual growth rate of aortic dilatation greater than 1.01 mm/year (Thanassoulis et al., 2008). Nevertheless, the results were different in an over 5-year prospective study which indicated that cusp fusion morphotypes did not increase the risk of aortic dilatation (Avadhani et al., 2015). There was no significant difference in the annual growth rate of the ascending aortic dilatation in asymptomatic BAV patients with R-L and R-N (0.47 mm/year and 0.46 mm/year, respectively; Avadhani et al., 2015). The aortic root growth rate was the highest in BAV patients with R-N morphotype and the smallest in BAV patients with type 0 (3.0 mm/year vs. 0.8 mm/year; Sophocleous et al., 2019).

Aortic dilatation can progress to aortic aneurysm or aortic dissection, both of which are the most common complications of BAV aortopathy. Data from a systematic review and meta-analysis study showed that the growth rate of ascending aortic aneurysm (diameter range, 35.5–56.0 mm) was 0.3 mm/year in TAV patients and 0.76 mm/year in BAV (Guo et al., 2018). Evidence implies that patients with BAV are more likely to have accelerated aortic dilatation and the onset of aortic complications earlier in life than those without BAV (Yasuda et al., 2003; Eleid et al., 2013). Aortic dissection is the most serious complication, and the overall in-hospital mortality rate could be as high as 19% (Reutersberg et al., 2019). The prevalence of aortic dissection among BAV patients is reportedly 0.31–0.50%, a figure that is not incredibly high in and of itself, but an eight-times higher risk than those in the general population (Michelena et al., 2011; Kong et al., 2017). The natural history of BAV aortopathy has not been well elucidated, except for a few observational studies. In a retrospective cohort study of 384 patients without aneurysms at baseline, after 15 ± 6 years of follow-up, 49 BAV patients developed aneurysms. The incidence of aneurysm in BAV was 84.9 cases per 10,000 patient-years, which is an 86-fold higher risk than the general population (Michelena et al., 2011). After aneurysm diagnosis, the 15-year risk for aortic dissection was 7% (Michelena et al., 2011).

Aortic diameter and growth rate are important factors for clinical strategies regarding either surgical intervention or conservative observation in patients with BAV. The larger the baseline aortic diameter, the faster the aortic dilatation (Park K. H. et al., 2017). However, no typical linear correlation between baseline aortic size and aortic dilatation has been observed (Detaint et al., 2014). Interestingly, smaller baseline aortic diameters indicate an increased risk of dissection at smaller diameters in BAV patients with insufficiency (Girdauskas et al., 2015). Studies have suggested that aortic valve dysfunction (AR or AS) accelerates aortic dilatation in patients with BAV (Park K. H. et al., 2017; Evangelista et al., 2018). However, Avadhani et al. (2015) indicated that aortic valve dysfunction was not associated with faster aortic dilatation. The risk factors associated with aortic dilatation are not only complex, but sometimes vary widely among studies. Furthermore, most of the study groups varied in terms of the initial aortic diameter, age, imaging technique used, and follow-up time, making comparisons difficult to determine risk factors.

Coarctation of the aorta (CoA) may accelerate progression of aortic dilatation in other aortic segments, especially for the ascending aorta and post-CoA, resulting in fatal complications, including aortic aneurysm, aortic dissection, and aortic rupture (Erbel et al., 2014). A history of CoA was reported to be a risk factor for ascending aortic dilatation and type A aortic dissection (Oliver et al., 2009; Eleid et al., 2013; Zhao et al., 2018). The group with a severe degree of CoA had a significantly larger ascending aorta and post-CoA diameter than the group with a mild degree of CoA (Zhao et al., 2018). The current consensus is that BAV patients with a history of CoA (cBAV) should have a lower threshold for preventative aortic surgery (Erbel et al., 2014). However, the findings of Davies et al. (2007) challenged the contemporary intervention strategy for cBAV patients. They pointed out that CoA is not a risk factor in BAV patients. CoA does not increase the risk of aortic dissection, preventative aortic surgery, aortic dilatation, or faster aortic growth in BAV patients (Duijnhouwer et al., 2020). It is worth mentioning that Davies et al.’s (2007) study encouraged the exploration of prognostic differences in BAV patients with or without CoA but lacked the exploration of effects of risk factors of CoA severity and CoA repair history; this requires more research in the future.

## Pathophysiology

### Genetic Predisposition

Bicuspid aortic valve is considered to be inherited in an autosomal dominant manner with incomplete penetrance and has a higher aneurysm susceptibility. Current studies generally agree that aortic aneurysms in BAV and TAV patients originate as a result of different molecular, cellular, and genetic mechanisms. Genetic and hemodynamic basis are the two major etiologies of aortic disease in BAV aortopathy, and despite being controversial, both have valid evidence. Progressive aortic dilatation can occur in BAV patients with normally function or in BAV patients after aortic valve replacement, all which suggesting inherent lesions of the arterial wall or genetic etiology.

The genetic basis of BAV has been extensively studied in recent years, but only a few pathways have been identified. A genome-wide association study revealed that BAV and/or associated cardiovascular malformations exhibit linkage to chromosomes 18q, 5q, and 13q (Martin et al., 2007). Mutations in the *ACTA2* gene, which encodes for smooth muscle α-actin, may cause thoracic aortic aneurysm and/or BAV (Guo et al., 2007). The *SMAD6* gene variation is associated with thoracic aortic aneurysm in patients with non-syndrome BAV (Galvin et al., 2000; Gillis et al., 2017; Luyckx et al., 2019). However, only a few of the *SMAD6* variants presented as BAV were accompanied by thoracic aortic aneurysm, while most of the variants presented variable clinical phenotypes (Luyckx et al., 2019; Park et al., 2019). *NOTCH1* has been identified as a candidate gene associated with BAV. In addition to a few rare syndromic forms of BAV, the *NOTCH1* missense variants increase aneurysm susceptibility (Harrison et al., 2019a). The deregulation of the *NOTCH1* signaling pathway in BAV and early ascending aortic aneurysm formation can be detected in both tissue and circulating levels (Balistreri et al., 2018). A recent study confirmed that *ROBO4* is involved in BAV formation and aneurysm development (Gould et al., 2019). In addition, *GatA5*, *TGFBR1/2*, *FBN1*, *ADAMTSL1*, *ADAMTS-4*, and *NOS3* are also candidate genes associated with BAV aortic disease (Arrington et al., 2008; Folkersen et al., 2011; Laforest et al., 2011; Ren et al., 2017; Rocchiccioli et al., 2017; Vorkapic et al., 2017; Peterson et al., 2020). However, genetic mutations involved both in the formation of BAV and the development of aneurysms remain unknown. Questions regarding how genes are involved in the formation of BAV aneurysms, or whether the genes that cause BAV disease are necessary to subsequent aneurysm formation are still unanswered. We have only glimpsed the tip of the iceberg and still need to explore these aspects further. Genes that have been associated with BAV aortopathy are summarized in Table 1. It should be noted that no single gene model can fully explain BAV or BAV aortopathy, meaning that BAV aortic disease might involve the genetic structure of very complex interactions among several genes.

**TABLE 1:** Summary of genes associated with BAV aortopathy.

Genes	References
ACTA2	Guo et al., 2007
SMAD6	Galvin et al., 2000; Gillis et al., 2017; Luyckx et al., 2019
NOTCH1	Harrison et al., 2019a
ROB04	Gould et al., 2019
GatA5	Laforest et al., 2011
TGFBR1/2	Arrington et al., 2008; Rocchiccioli et al., 2017
FBN1	Folkersen et al., 2011
ADAMTSL1	Vorkapic et al., 2017
ADAMTS-4	Ren et al., 2017
NOS3	Peterson et al., 2020
chromosomes 18q, 5q, and 13q	Martin et al., 2007

### Histopathological Features of the Aorta

The histopathologic features of ascending aortic aneurysms in humans are degeneration or cystic necrosis of the intermediate layer of the aorta. Vascular smooth muscle cells (SMCs) and the extracellular matrix (ECM) are important components of the intermediate layer. The ECM is characterized by its composition of elastic fibers, collagen, and proteoglycan, which affects the migration and proliferation of SMCs (Lu and Daugherty, 2017). Abnormalities either on ECM or SMCs could threaten the homeostasis of the aortic wall (Lu and Daugherty, 2017). Without a doubt, abnormal SMCs and ECM degradation are important markers of aortic aneurysm formation (Parai et al., 1999). The histological presentation of BAV aneurysms is complex. The literature has shown that the aortic histopathological changes of BAV are more serious than that of TAV; specifically, cystic medial necrosis, elastic rupture, and SMCs loss in the ascending aorta are more serious (De Sa et al., 1999; Schmid et al., 2004; Chen et al., 2020). A trend of most studies is that compared with TAV-associated aneurysms, aortic histopathological changes of BAV-associated aneurysms are relatively fewer and less severe (Matthias Bechtel et al., 2003; Collins et al., 2008; Balistreri et al., 2013; Stern et al., 2019).

Matrix metalloproteinases (MMPs) and endogenous inhibitors (matrix metalloproteinase organization inhibitors, TIMPs) are in dynamic balance under normal physiological conditions. Once an imbalance in the expression of MMPs and TIMPs sets in, the degradation of the ECM becomes abnormal. In BAV patients with non-dilated aorta, as study found that MMP-2 expression increased and MMP-14 expression decreased significantly vs. normal aorta (Forte et al., 2017). Another study indicated that only the expression of MMP-2, but not that of MMP-9, increased; further, only TIMP-1 expression decreased in thoracic aortic aneurysms (TAA) with BAV compared to TAV (Rabkin, 2014). Research has also suggested that in less-dilated aorta, the expression of TIMP-1, -2, and -3 were significantly decreased, while the activity of MMP-2 was significantly increased; whereas, in severe-dilated aortas, TIMP expression increased and MMP-2 activity declined to baseline levels (Wu et al., 2016). The reason for the inconsistent results in these studies may be attributed to different sample sources tested or the heterogeneity of BAV aortic disease. Fibrillin 1 is an important bridge for SMC adherence to the ECM, and defects in its expression will stimulate the activation of MMPs to degrade the ECM, thereby reducing the structural integrity of the vessel wall. Fibrillin-1 content was reportedly reduced in BAV aortas compared to TAV aortas (Fedak et al., 2003; Rueda-Martínez et al., 2017).

Abnormal SMC differentiation leads to progression of vascular pathology, and prematurely aged SMCs are associated with a destructive senescence-associated secretory phenotype (SASP; Ailawadi et al., 2009; Grewal et al., 2014). Balint et al. (2019) reported a particular predisposition to premature aging of SMCs in BAV aortopathy and found a large number of senescent SMCs in aortic tissue in the early stage of BAV. SMCs in TAV- and BAV-associated TAA were also different in terms of proliferation and metabolism (Blunder et al., 2012). SMCs showed a senescent state in BAV-TAA and a proliferative state in TAV-TAA (Blunder et al., 2018). The *NOTCH1* signaling pathway and osteogenic induction response can affect the differentiation and function of SMCs (Ignatieva et al., 2017). Defective *NOTCH1* signaling drives increased vascular SMC apoptosis and contractile differentiation in BAV aortopathy. The latter resulted in ECM degradation and the inability for tissue repair (Harrison et al., 2019b). Myocardin is a master regulator of *SMC* gene expression and differentiation (Wang et al., 2003), and its decreased expression has been reported in dilated aorta samples from BAV patients (Forte et al., 2013). PDE5 is a cGMP hydrolase that is highly expressed in aortic SMCs, and its lack of function may affect the development of the aortic wall (Cesarini et al., 2019).

The endothelial mesenchyme transition (EndoMT) refers to the loss of endothelial/epithelial cell features and acquisition of mesenchyme traits, which are indispensable during development (Kovacic et al., 2012; Lamouille et al., 2014). Improper EndoMT regulation during the embryogenesis of BAV and ascending aorta and postembryonic EndoMT state deterioration were important causes of ascending aortic aneurysm (Maleki et al., 2016). A recent study indicated that targeted silencing of *ROBO4* or mutant *ROBO4* expression in ECs led to impaired endothelial barrier function and EndoMT, making individuals vulnerable to BAV-TAA (Gould et al., 2019). Activation of *NOTCH* signaling is one of the important signaling pathways to induce EndoMT. *NOTCH1* signaling was down-regulated and *NOTCH*-dependent EndoMT failed to activate in aortic ECs in BAV-TAA (Kostina et al., 2016). EndoMT is regulated by several complex extracellular signaling pathways. In addition to the signaling pathways induced by specific genes, transforming growth factor-β (TGF-β) signaling pathways, DNA methylation, and some microRNAs are also involved in the regulation of EndoMT in BAV patients with aortic aneurysm (Forte et al., 2017; Björck et al., 2018; Mohamed, 2019).

### Epigenetics

Epigenetics refers to changes in gene expression that are not caused by changes in the gene sequence. The study of epigenetic signal expression in BAV aortic disease is still in its infancy, and current studies mainly focus on DNA methylation and microRNAs (miRNAs). Pan et al. (2017) performed DNA methylation bead array analysis on ascending aorta samples and found for the first time that both aortic dissection and BAV with aortic aneurysmal dilatation were characterized by DNA methylation loss at non-CpG sites. Compared with non-CpG sites, these two diseases showed distinct DNA methylation landscapes at CpG sites; in particular, BAV primarily manifested as hypermethylation of EZH2 targets (Pan et al., 2017). Further study indicated DNA methylation in aortic intima-media tissue samples of BAV were associated with EndoMT disorder (Björck et al., 2018).

miRNA are reportedly involved in the development and progression of BAV aortic dilatation. Circulating mir-17, mir-20a, and mir-106a levels were significantly negatively and linearly correlated with proximal aortic diameter in BAV patients with normal aortic root dimensions (Girdauskas et al., 2020). Analysis of the entire miRNAome in TAA tissue from BAV and TAV patients indicated that mir-424-3p and mir-3688-3p were significantly down-regulated in BAV patients compared to TAV patients (Borghini et al., 2017). Wu et al. (2016) collected samples of severely dilated and non-dilated aortic tissue from 12 patients with BAV and evaluated gene and protein expression levels in paired tissue samples from the same patient. In the early stage of aortic dilatation, the up-regulation of mir-17 expression strongly inhibits TIMPs targets, thereby, increasing the activity of MMP-2. Then, the activated MMP-2 contributes to progressive ECM destruction, aortic dilatation, and aneurysm formation. In severely dilated aortas, the expression of mir-17 was reduced, resulting in increased TIMP expression and a decline of MMP-2 activity to baseline levels. The important role of mir-17 in regulating the TIMP-MMP pathway was further confirmed in lab-grown aortic SMCs to provide new insights into the mechanisms of BAV aortic dilatation (Wu et al., 2016). For BAV patients with non-dilated aorta (aortic diameters, <40 mm), the mir-200 family showed low expression, whereas the mir-200 target gene and ZEB1/ZEB2 transcription factors were highly expressed (Maleki et al., 2019). Lower expression of circulating mir-145 in BAV patients with aortic root dilatation (aortic root diameter, ≥40.0 mm) is associated with the presence of rare NOTCH1 variants (Girdauskas et al., 2018). Although miRNAs are valuable biomarkers, it appears that the measurement of a single miRNA analyte cannot predict aneurysmal disease, and there may be complex interactions among the analytes (Albinsson et al., 2017; Maleki et al., 2019).

## Hemodynamics

Hemodynamic characteristic changes caused by valve malformations have long been considered a cause of BAV aortopathy in addition to genetic predisposition. As one of the major complications of BAV, AS is a known independent risk factor for ascending aortic dilatation/aneurysm. It is an important basis of hemodynamic theory that patients with AS are prone to aortic dilatation after stenosis. The hemodynamic mechanism of BAV aortopathy is both an old and novel theory. Previous researchers could only provide inferences from theories and observations due to outdated imaging technology. In recent years, advanced imaging technology has promoted the in-depth exploration and improvement of hemodynamic mechanisms. In particular, four-dimensional flow magnetic resonance imaging (4D flow MRI) technology affords direct, non-invasive, and real-time measurement of aortic three-dimensional flow (Markl et al., 2012). At the same time, 4D flow MRI can comprehensively display important parameters, such as flow direction, velocity, and wall shear stress (WSS; Markl et al., 2012). Moreover, the accuracy of 4D flow MRI in measuring the peak flow velocity of the aortic valve was similar to that of the ultrasonic Doppler technique (Rose et al., 2016).

Disturbance of aortic hemodynamics leads to non-uniform WSS, which is considered one of the mechanisms leading to BAV aortopathy and local aortic wall lesions (Grewal and Gittenberger-De Groot, 2019). Flow pattern and local WSS distribution in the aorta were changed in BAV patients without aortic aneurysm or even with normal valve function (Hope et al., 2010; Garcia et al., 2016). Further, the valve morphologic phenotype of BAV was the most important factor influencing WSS distribution, systolic flow eccentricity, and phenotypes of BAV aortopathy (Mahadevia et al., 2014). Different valve morphology showed different outflow jet direction and a velocity profile matched with regional WSS patterns. The velocity profile and maximum axial WSS of BAV R-L were uniformly distributed, and flow toward the anterior and right-anterior aortic walls (Mahadevia et al., 2014). In contrast, BAV R-N showed higher variable profiles with main posterior output flow at the STJ that shifted to the anterior or right-anterior walls in the mid and distal ascending aorta (Mahadevia et al., 2014). The eccentric outflow jet and WSS resulted in a greater tendency of proximal aortic dilatation in BAV R-L patients and a greater tendency of dilatation of the ascending aorta and proximal aortic arch in BAV R-N patients (Mahadevia et al., 2014; Rodríguez-Palomares et al., 2018). Interestingly, one study by Van Ooij et al. (2017) showed that regional WSS patterns were very similar in BAV and TAV patients with significant AS (moderate and severe).

**FIGURE 2:**
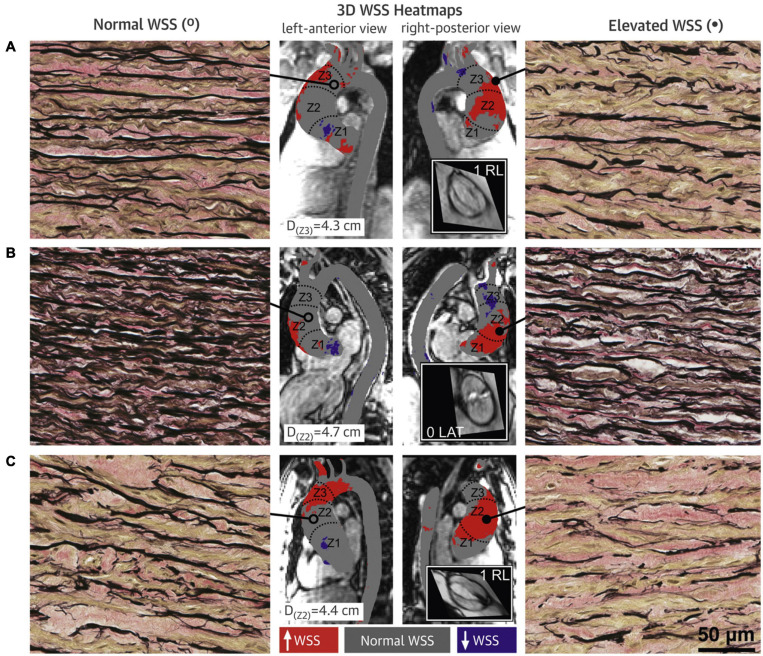
Wall shear stress heat maps and elastin fiber staining of BAV aorta wall. Aortic wall (**A–C** denote three different BAV aortas) from regions of high WSS (right panels; closed circles) had fewer elastin fibers (black) that were thinner and farther apart compared with regions with normal WSS (left panels; open circles) in the same human aortas (40 × magnification). Samples were collected from zone 1, 2, or 3, and from either the greater curvature, lesser curvature, anterior wall or posterior wall; accompanying diameters for tissue collection sites are shown. Gray denotes normal WSS, within the 95% confidence interval, compared with a healthy tricuspid aortic valve population; red and purple denote elevated and depressed WSS, respectively. Insets show steady-state free precession images of the aortic valve and Sievers valve phenotype. 3D, three-dimensional. Adapted with permission (Guzzardi et al., 2015).

Studies about the associations between hemodynamic stresses and tissue histopathology make the hemodynamic theory more convincing. Atkins et al. (2014) used fluid structure interaction (FSI) simulation in an *ex vivo* model of the porcine aorta model subjected to both valvular flows to predict WSS environments generated in the convex region of a TAV and BAV ascending aorta. Normal porcine aortic tissue was exposed to the corresponding WSS environments for 48 h in a cone-and-plate bioreactor. FSI simulation revealed the existence of larger and more unidirectional WSS in BAV than TAV ascending aorta convexity. Relative to the TAV ascending aorta WSS treatment, expression of MMP-2 and MMP-9 and activity of MMP-2 were increased after BAV ascending aorta WSS treatment (Atkins et al., 2014). This confirmed the sensitivity of aortic medial tissue to WSS abnormalities and provided compelling support for the important role of hemodynamic stresses in BAV aortopathy (Atkins et al., 2014). Guzzardi et al. (2015) performed 4D flow MRI on patients undergoing aortic dissection and preoperatively identified the location of high and normal WSS regions. Then, the aorta wall specimens at the corresponding locations were collected during surgery and compared for histopathological examinations. Compared with the area of normal WSS, ECM dysregulation was more severe in the high WSS regions with greater medial elastin degradation and increased expression of TGFβ-1, MMP-1, MMP-2, MMP-3, and TIMP-1 observed (Guzzardi et al., 2015; Figure 2). The significance of Guzzardi et al.’s (2015) study was that they provided reliable evidence for the hemodynamic theory of BAV aortopathy.

The majority of current 4D flow MRI studies have primarily focused on the qualitative and quantitative evaluations of hemodynamic characteristics and stress, which can help to identify those at risk for developing ascending aortic aneurysm. The changes in flow dynamics associated with aortic valve morphology might precede the onset of aortic disease and may drive aortic remodeling and dilatation over time (Piatti et al., 2017); however, large-scale clinical longitudinal studies for validation of these results are still lacking and need further exploration in the future.

## Clinical Significance

### Imaging Diagnosis

2D-TTE is the most commonly used and initial imaging modality for diagnosing BAV (Goldstein et al., 2015). In general, 2D-TTE can fully display aortic valve configuration, as well as the aortic root (aortic annulus, sinuses of Valsalva, STJ) and proximal segment of the tubular ascending aorta. However, 2D-TTE is more limited for measuring the middle and distal segments of the tubular ascending aorta, the aortic arch, and the distal part of the descending thoracic aorta. If diagnosis of BAV malformation was clear or highly suspected by 2D-TTE, both the aortic root and the ascending thoracic aorta should be examined as much as possible to determine evidence of aortic dilatation (Hiratzka et al., 2010b). When 2D-TTE does not adequately show any aortic segments, cardiac MRI or electrocardiographically gated computed tomographic angiography (CTA) is necessary for a comprehensive assessment of the entire thoracic aorta (Borger et al., 2018).

The importance of multimodal imaging to monitor aortic diameters has been widely recognized, but there is no consensus on the best measurement approach (Goldstein et al., 2015). Societal consensus for aortic measurement by CTA or MRI is not currently available, except for echocardiography (Erbel et al., 2014). Measurement of the aortic annulus from inner-to-inner edges (I-I) in the middle systole phase and ascending aorta from leading edge to leading edge (L-L) method at the end of the cardiac diastolic phase have been the standard measuring options for TTE in adults (Goldstein et al., 2015; Lang et al., 2015). The aortic root and ascending aortic diameters measured by TTE using the L-L method have shown excellent consistency and reproducible values compared to CTA and MRI with the I-I method (Rodríguez-Palomares et al., 2016; Tretter and Mori, 2019). The irregular anatomic geometry of aortic roots and sinuses is a characteristic of BAV patients (Shibayama et al., 2014; Vis et al., 2019), which widens the measurement difference between MRI and TTE, especially in BAV patients with asymmetric aortic roots and/or with right and non-coronary cusps fusion (Vis et al., 2019). Available data using the CTA outer wall-to-outer wall (O-O) method to measure the sinus of aorta showed that the correlation and consistency were best with TTE parasternal short axis (SAX) by mid-diastolic L-L method and TTE parasternal long axis (LAX) by end-diastolic L-L method (Park J. Y. et al., 2017).

The latest guidelines (Borger et al., 2018) suggest that a diameter of the ascending aorta measured by echocardiography ≥ 45 mm should be further examined with MRI or CTA. If initial measurements are discrepant (>2 mm), CTA or MRI is recommended as the preferred technique for the measurement of aortic follow-up (class I/C). Reimaging is recommended every 3–5 years if the diameter of the aorta is normal (<40 mm; Borger et al., 2018). If the diameter of the tubular ascending aorta or root is 50–54 mm, reimaging will be recommended at least every 12 months (class I/C; Borger et al., 2018). If the diameter of the tubular ascending aorta or root is 40–49 mm, reimaging is recommended the next year (class I/C; Borger et al., 2018). If the diameter of the ascending aorta or root is 40–49 mm, a further imaging examination is recommended at 12 months (class I/C; Borger et al., 2018). This is the exclusive consensus on BAV aortopathy regarding the interval of monitoring imaging, which indicates that TTE is the baseline imaging modality for routine screening and interval monitoring, while MRI and CTA assessment are the gold standards. More research is needed in the future to explore the yields and benefits of such protocols.

All imaging techniques have inherent limitations, so there is no perfect imaging technique. Echocardiography measurement depends on the operator’s experience and expertise to a certain extent. If the scanning section is oblique and/or the measured line is not perpendicular to the anterior and posterior walls of the aorta, it can easily lead to inaccurate measurement of the aorta. The aortic root may not only expand circumferentially but also longitudinally, which may also be the reason why TTE is not suitable for long-term interval follow-up. Although 2D TEE can provide sufficient image resolution to depict the anatomical structure, and 3D TEE may have added value, both are highly dependent on the operators’ quality; moreover, there are still limitations to accurate imaging of metal implants or calcifications. Compared to echocardiography, CTA can best identify thoracic aortic disease and is more suitable for monitoring after interventional or open-heart surgery. The sensitivity and specificity of MRI in the diagnosis of thoracic aortic disease have been demonstrated to be equal to or possibly greater than CTA and TEE (Erbel et al., 2014; Zhu et al., 2017). Both CTA and MRI cannot be performed in patients with contraindications to contrast agents, and MRI is not available in patients with metal implants or claustrophobia. For patients with aortic disease requiring repeated examination, MRI may be superior to CTA in terms of minimizing radiation exposure. For patients after cardiac surgery with metal implantation or those with financial constraints, MRI may not be as widely available with easily continuous monitoring as echocardiography or CTA.

### Intervention and Prognosis

In terms of clinical decision-making, there has always been a debate between active surgical intervention and conservative follow-up. The latest 2018 guidelines from the American Association for Thoracic Surgery (ATS) on BAV-related aortopathy define intervention indications (Borger et al., 2018). For BAV patients without risk factors, an ascending aorta/root diameter of 55mm is recommended as an indication for repair (class I/B; Borger et al., 2018). For patients with risk factors such as root phenotype or predominant aortic insufficiency, uncontrolled hypertension, family history of aortic dissection/sudden death, coarctation, and/or aortic growth > 3 mm/year, the recommended indication of ascending aorta/root repair is ≥50 mm of the aortic diameter (class IIa/B; Borger et al., 2018). For patients undergoing concurrent heart surgery, concomitant ascending aorta/root repair should be actively performed when the aortic diameter is 45 mm (class IIa/B; Borger et al., 2018). The indications for surgical intervention in the aortic root are influenced by the type of implanted valve. Among patients with moderate aortic root dilatation (4.5–5.0 cm) undergoing concurrent surgery, simultaneous complete aortic root replacement is justified for patients with mechanical valve selection (Borger et al., 2018). It is worth mentioning that these recommendations do not accurately stratify the prognostic risk of BAV patients based on family history, heart function, comorbidities, sex, age, and other factors. Most of the current guidelines for the establishment of intervention indications are based on retrospective studies, and there is a lack of large-scale and long-term longitudinal studies to provide support for indications.

The current guidelines recommend beta-blockers and angiotensin receptor inhibitor blockers (ARBs) for BAV patients with hypertension (Boodhwani et al., 2014; Borger et al., 2018). The basic mechanism of antihypertensive therapy is to reduce the mechanical pressure on the aneurysmal segment of the aorta by reducing arterial pressure. The underlying mechanism of therapeutic strategies may be the reason for the excessive TGF-β activation and signaling pathway in aortic dilatation and dissection observed (Neptune et al., 2003; Nataatmadja et al., 2013). Whether there is synergy between ARBs and beta-blockers, and whether the combination of the two is more effective than either ARBs alone or beta-blockers alone has not been clarified (Chiu et al., 2013; Lacro et al., 2014). Salt reduction and weight loss are also advocated as part of blood pressure control strategies (Borger et al., 2018). Although all evidence clearly supports the management of blood pressure in BAV patients, there are no consensus guidelines on the blood pressure management threshold of BAV patients with or without aortic dilatation (Wright et al., 2015; Borger et al., 2018).

Progression of BAV aortic dilatation can eventually lead to adverse aortic events (aortic aneurysm rupture or aortic dissection), and surgical intervention remains the basic route for the therapeutic management. Whether selective AVR or ascending aorta replacement can reduce the long-term risk of aortic adverse events in BAV patients is the key issue. Previous studies have suggested that AVR could not prevent progressive aortic dilatation in BAV. The aorta of BAV patients before AVR and after AVR showed similar progressive dilatation (Yasuda et al., 2003). After isolated AVR, patients with BAV were at higher risk of aortic dissection than TAV patients (Russo et al., 2002). Most current reports tend to suggest that postoperative progression of preserved native aortic segments progressed slowly. A retrospective study of 325 patients (BAV = 153, TAV = 172) after isolated AVR was performed by Girdauskas et al. (2014) After 15 years of isolated AVR, the risk of aortic dissection in patients with AS accompanied by ascending aortic dilatation of 40–50 mm was lower and no significant difference was noted between BAV and TAV patients (Girdauskas et al., 2014). Vendramin et al. (2016) found no progressive dilatation of the native aortic root in patients undergoing AVR and supracoronary ascending aorta replacement (AVR-SCAAR), regardless of whether the preoperative root diameter was <40 mm or >40 mm. In a retrospective cohort study of 1301 adult BAV patients by Kaneko et al. (2018), 683 BAV patients underwent AVR alone, while 618 BAV patients underwent AVR with aortic resection (AVR-AR). During a median follow-up of 6.6 years, no aortic dissection occurred in the AVR group or the AVR-AR group, and there was no significant difference in the outcome of reoperation or death (Kaneko et al., 2018).

It is still unclear if any drug therapy is effective in preventing adverse events in BAV aortopathy (i.e., aortic aneurysm and aortic dissection). Actually, the current guidelines of drug treatment were based on extrapolated evidence from populations with connective tissue disease (MFS; Boodhwani et al., 2014; Borger et al., 2018). Therapies that inhibit or attenuate TGF-β signaling pathway expression are recognized in patients with MFS. Beta-blockers, as well as ARBs, have been shown to be effective in preventing and slowing the progression of aortic dilation in patients with MFS (Baumgartner et al., 2010; Lindsay and Dietz, 2011; Emrich et al., 2019). ARBs have been shown to neutralize or attenuate TGF-β signaling pathway expression, such as irbesartan and losartan, and are as important as beta-blockers in slowing aortic dilatation and reducing aortic dilatation rates (Cohn et al., 2007; Lindsay and Dietz, 2011; Emrich et al., 2019). However, a recent study has shown that the mechanism of losartan’s inhibition of aortic root dilatation in MFS is the independent activation of endothelial function by angiotensin II receptor type 1 (Sellers et al., 2018). However, whether antihypertensive drugs can reduce the incidence of long-term aortic adverse events or the rate of aortic surgery remains to be elucidated. Whether the therapeutic effects of the strategies extrapolated from MFS can be replicated on BAV remains to be further explored (Allen et al., 2016). Compared to thoracic aortic aneurysms, antihypertensive therapy (β-blockers or ACE inhibitors) cannot slow the progression of abdominal aortic aneurysms (Lindeman and Matsumura, 2019). Statins are not currently included in the guidelines, but studies have shown that statin use can reduce the odds of ascending aortic dilatation in BAV (Goel et al., 2011; Taylor et al., 2016; Sequeira Gross et al., 2018).

Regardless of whether the aorta was dilated, aortic elasticity in BAV patients was significantly lower than that in patients with TAV (Goudot et al., 2019a). Theoretically, reduced aortic elasticity can increase the left ventricular afterload to some extent (Ohyama et al., 2016). Some studies have suggested that aortic stiffness is associated with early left ventricular dysfunction in patients with BAV (Lee et al., 2015; Ohyama et al., 2016). However, there have also been studies that have shown no association between aortic stiffness and left ventricular dysfunction (Kurt et al., 2012; Weismann et al., 2016). BAV disease might not be confined to the aortic valve or aorta but may also involve the myocardium due to a common genetic basis of variation. Nevertheless, considering the role of ventricular-arterial coupling in heart failure (Ikonomidis et al., 2019), the effect of BAV aortopathy on left ventricular function still deserves further investigation.

### Special Populations

Aortic disease is one of the important causes of maternal indirect death (Cantwell et al., 2011). Most scholars believe that pregnancy is a major risk factor for adverse aortic events (Yuan, 2013; Toprak et al., 2020). It may be that the hyperdynamic and hypervolemic cardiovascular state in pregnancy that increases the stress and shear force of the aortic wall has significant effects on the aorta (Cox et al., 2014; Bons and Roos-Hesselink, 2016). Another possible effect of pregnancy is that pregnancy-induced alterations in the hormonal profile may lead to significant changes in aortic structure and integrity (Nolte et al., 1995). It should be noted that, in addition to pregnancy, connective tissue diseases such as BAV and MFS themselves, are also risk factors for aortic dissection (Hiratzka et al., 2010a; Michelena et al., 2011). Interestingly, a recent retrospective study showed that pregnancy only increases the immediate aortic risk of MFS patients, but not that of BAV patients (Toprak et al., 2020). Further, pregnant women with MFS have a higher risk of aortic dissection or proximal aortic surgery than BAV alone (Toprak et al., 2020). Therefore, whether this management strategy for women with MFS can be extrapolated to women with BAV is limited by the absence of reference research data. Although previous pregnancies cannot increase long-term risk of adverse aortic events (Toprak et al., 2020), pre-pregnancy evaluation and post-pregnancy management of pregnant women with cardiac disease by professionals is still necessary (Hiratzka et al., 2010a; Borger et al., 2018). For female patients with MFS who plan to become pregnant, ACCF/AHA guidelines recommend a surgical threshold of 40 mm for the aortic diameter (Hiratzka et al., 2010a), while the ESC guidelines recommend a threshold of 45 mm (Erbel et al., 2014). For pregnant women at high risk of aortic dilatation or aortic dilatation with or without hypertension, the recommended preventative treatment includes beta-blockers to control the heart rate and reduce shear stress in the aortic wall (Hiratzka et al., 2010a).

Theoretically, the adrenaline rush and increased hemodynamic load of the heart during strenuous exercise may increase the stress and shear on the walls of the aorta (Braverman et al., 2015). High-intensity endurance training may promote aortic remodeling (Churchill et al., 2020). Nonetheless, it is unclear whether restricting physical activity limits the odds or risk of aortic dilatation or dissection. In a cross-sectional study of 442 older masters-level athletes, 94 (21%) had ascending aorta sizes measuring ≥ 40 mm (Churchill et al., 2020). Further, aortic diameter was significantly greater in athletes compared with the general population (Churchill et al., 2020). The 2015ACC/AHA consensus opinion recommends restricted physical activity and complete avoidance of any competitive sports, if the aortic diameter of athletes with BAV reaches 45 mm (Braverman et al., 2015).

A recent consensus on BAV slightly lowered the threshold for exercise restriction, by setting the aortic diameter threshold at 45 mm for BAV patients, who should avoid high-intensity physical or competitive exercise (Borger et al., 2018). One meta-analysis showed that the aortic root diameters in athletes were larger than those of non-athletic individuals (Iskandar and Thompson, 2013). One follow-up study of elite BAV athletes suggested that high-intensity training and sports competition did not aggravate BAV symptoms during elite athletes’ careers (Boraita et al., 2019). Elite BAV athletes with mild-to-moderate aortic dilatation did not require restrictions regarding exercise intensity and experienced no adverse consequences (Boraita et al., 2019). Another recent study pointed out that stricter enforcement of current guidelines would limit more than a third of school-age children and young adults with BAV from participating in some form of competitive sport during their school years (Baleilevuka-Hart et al., 2020). More research is needed in the future to better understand the true impact of exercise on the progression of BAV aortic disease and adverse cardiac events. Excessive restriction of exercise may lead to social discrimination and increase the psychological burden of patients (Braverman et al., 2015). Therefore, we advocate that BAV patients or athletes should perform moderate exercise according to their own abilities, which is beneficial to both physical and mental health and insist on regular imaging follow-up. In addition, the incidence of aortic dilatation in first-degree relatives of BAV patients is higher than that of the general population (Biner et al., 2009; Galian-Gay et al., 2019). The first-degree relatives of BAV patients should undergo screening and evaluation by echocardiography, especially if the proband have a dilated aorta (Biner et al., 2009; Borger et al., 2018).

## Future Perspective

Bicuspid aortic valve aortic dilatation is heterogeneous in terms of clinical features, epidemiology, and phenotype patterns. Genetic predisposition and hemodynamic effects are well-recognized as the two major mechanisms of BAV aortic dilatation pathogenesis. It may be that complex interactions of such elements reflect distinct phenotypes of BAV aortic dilatation. Emerging genetics, biomarkers, and advanced imaging modalities should be combined to gain a better understanding of the mechanisms underlying the development and progression of bicuspid aortopathy. The efficacy of drug therapy for aortic dilatation of BAV is still uncertain. Due to the genetic basis of BAV disease, gene targeted therapy might be a research trend in the future. Judicious control of blood pressure through pharmacological and non-pharmacological measures is recommended. To establish a risk prediction model that can accurately evaluate aortic dilatation or dissection by combining hydrodynamic imaging indicators and other parameters like aortic elasticity is worth expecting.

**FIGURE 3:**
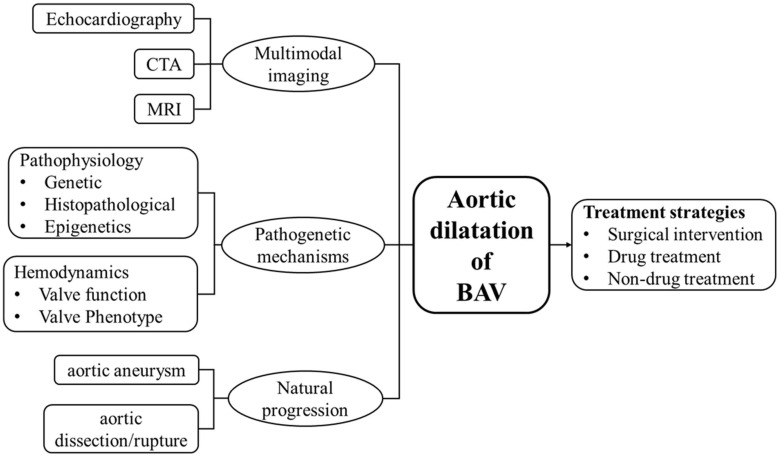
In order to better understand the status of aortic dilatation in patients with BAV and the direction of further research, a brief diagram is provided. Includes natural progression (aortic aneurysm and aortic dissection/rupture), pathogenetic mechanisms (pathophysiology and hemodynamics), multimodal imaging technology (echocardiography, CTA, MRI), and treatment strategies.

We hope that future research can combine natural disease progression, pathogenetic mechanisms and multimodal imaging technology to define the risk of BAV aortopathy at a highly individualized level, which would assist in the development of individualized treatment strategies (Figure 3). Although surgical intervention remains the basic route for the therapeutic management of aortic dilatation in BAV patients, the time period between diagnosis and need for surgical intervention is generally long, which represents a great potential value in preventative medical treatment. The evidence-based natural history of BAV aortic dilatation helps in precisely improving identification of patients who are most likely to benefit from early intervention, as well as improving the surgical indications and prognosis of those who need a conservative wait-and watch approach.

## Author Contributions

JW and WD contributed equally to this work, performed the literature review, and drafted the manuscript. QL and YL contributed to the revision and editing of the manuscript. TL collected the manuscript. MX revised and critically appraised the manuscript for intellectual content. All authors read and approved the final manuscript.

## Conflict of Interest

The authors declare that the research was conducted in the absence of any commercial or financial relationships that could be construed as a potential conflict of interest.
